# Contemporary Ventilation Strategies And Adjunctive Therapies For Acute Respiratory Distress Syndrome (Ards)

**DOI:** 10.1186/2197-425X-3-S1-A6

**Published:** 2015-10-01

**Authors:** EH Duan, NK Adhikari, L Hand, F D'Aragon, P Austin, Q Zhou, W Alhazzani, DJ Cook, ND Ferguson, MO Meade, Canadian Critical Care Trials Group

**Affiliations:** McMaster University, Hamilton, Canada; University of Toronto, Toronto, Canada

## Introduction

Over the past 5 years, several interventions have been shown in randomized clinical trials (RCTs) to reduce mortality in patients with ARDS. The current use of these interventions in clinical practice, along with those of unproven benefit, is unknown.

## Objectives

To determine which ventilation strategies and adjunctive treatments clinicians use for moderate-severe ARDS, and which ones they use as rescue therapy in refractory hypoxemia.

## Methods

We conducted a 12-month observational study (2014/03-2015/02) in 23 Canadian and 1 Saudi Arabian intensive care units (ICUs) that had participated in the OSCILLATE RCT of high frequency oscillation (HFO). We included mechanically ventilated adults with moderate-severe ARDS (Berlin criteria), defined by PaO_2_/FiO_2_ ≤200 with positive end-expiratory pressure (PEEP) ≥5 cmH_2_O, and who also required a FiO_2_ ≥0.50. Consecutive patients were prospectively identified and we collected baseline demographics, daily ventilation settings and adjunctive therapies for ARDS up to ICU day 28, and hospital outcomes.

## Results

These preliminary results include data from 533 patients. On day 1 of ARDS diagnosis, the most common ventilator modes were pressure control (n = 222, 42%) and volume control (n = 123, 23%). The mean FiO_2_ was 0.74 (SD = 0.21), tidal volume 7.4 ml/kg (SD = 2.2) predicted body weight (PBW), plateau pressure 26 (SD = 7) cmH_2_O and PEEP 11 (SD = 4) cmH_2_O. One-third of patients (155/466) were ventilated with tidal volume >8ml/kg PBW on day 1. in all patients, the most common respiratory adjuncts used were neuromuscular blocking agents (NMBA) (n = 204, 38%), corticosteroids (n = 139, 26%), pulmonary vasodilators (n = 88, 17%), prone positioning (n = 48, 9%), HFO (n = 24, 5%) and extracorporeal life support (ECLS) (n = 23, 4%). Among 82 patients with refractory hypoxemia, defined as a sustained PaO_2_ ≤60 mmHg on FiO_2_ 1.0, use of adjuncts increased: NMBA (n = 52, 63%), pulmonary vasodilators (n= 39, 48%), corticosteroids (n = 26, 32%), prone positioning (n = 21, 26%), HFOV (n = 14, 17%) and ECLS (n = 10, 12%). Median length of mechanical ventilation was 9 (IQR = 4, 17) days and ICU length of stay was 11 (IQR = 6, 20) days. ICU mortality was 46% (225/491) overall, and higher (63%, 49/78) among patients with refractory hypoxemia. Withdrawal of life support preceded 36% of deaths (n = 80).

## Conclusions

Clinicians generally utilized low tidal volume ventilation, although in many patients the recommended threshold was exceeded. Initial PEEP levels were lower than those used in high PEEP arms of several RCTs. HFOV was used infrequently in these centres. The most common respiratory adjunct was NMBAs. Despite stronger RCT evidence to support prone positioning, it was employed infrequently.

## Grant Acknowledgment

Funded by the Canadian Institutes of Health Research.

